# The Influence of Hafnium Doping on Density of States in Zinc Oxide Thin-Film Transistors Deposited via Atomic Layer Deposition

**DOI:** 10.1186/s11671-017-1852-z

**Published:** 2017-01-23

**Authors:** Xingwei Ding, Cunping Qin, Jiantao Song, Jianhua Zhang, Xueyin Jiang, Zhilin Zhang

**Affiliations:** 1 Key Laboratory of Advanced Display and System Application, Ministry of Education, Shanghai University, 149 Yanchang Road, Jingan District, Shanghai, 200070 People’s Republic of China; 2 School of Mechatronics and Automation, Shanghai University, Shanghai, 200072 China; 3 Department of Materials Science, Shanghai University, Shanghai, 200072 China

## Abstract

Thin-film transistors (TFTs) with atomic layer deposition (ALD) HfZnO (HZO) as channel layer and Al_2_O_3_ as gate insulator were successfully fabricated. Compared with ZnO-TFT, the stability of HZO-TFT was obviously improved as Hf doping can suppress the generation of oxygen related defects. The transfer characteristics of TFTs at different temperatures were also investigated, and temperature stability enhancement was observed for the TFT with Hf doping. The density of states (DOS) was calculated based on the experimentally obtained *E*_a_, which can explain the experimental observation. A high-field effect mobility of 9.4 cm^2^/Vs, a suitable turn-on voltage of 0.26 V, a high on/off ratio of over 10^7^ and a steep sub-threshold swing of 0.3 V/decade were obtained in HZO-TFT. The results showed that temperature stability enhancement in HfZnO thin-film transistors are attributed to the smaller DOS.

## Background

ZnO-based thin-film transistors (TFT) have recently attracted a great deal of attention owing to their potential application in active matrix liquate crystal display (AMLCD) and active matrix organic light emitting diodes (AMOLED) [1–3]. As an important part of TFTs, channel layers play a crucial role in TFT performance. ZnO-based single channel layer or double channel layer, such as HZO [4], IGZO [5], and IZO/IGZO [6], has been investigated for use in TFTs because of their high mobility. Among those ZnO-based TFTs, amorphous indium-gallium-tin oxide (IGZO) TFTs are regarded as the most promising devices as indium and gallium have excellent lattice matching with ZnO and gallium could suppress carrier generation via oxygen vacancy formation in IGZO. Recently, LG Display has released a 55 in, full high-definition (FHD) OLED television which utilizes an IGZO TFT active matrix [7]. However, materials such as indium (In) and gallium (Ga) have some disadvantages, including toxicity, element scarcity, and indium extraction in hydrogen plasma [8]. Specifically, the instability of ZnO-based TFTs is still a problem hard to solve, as ZnO-based TFTs usually contain defects in the active channel layer and deep-level defects in the channel/insulator interface [9]. To obtain highly stable Zn-based oxide TFTs, many studies have been devoted to the development of robust active layers by the incorporation of metal cations (such as Ga, Al, Hf, and Si) based on sputtering method or spin-coating method [10–14], with the expectation to reduce the density of defects such as oxygen vacancies. There are few reports on adopting ALD method to realize doping in Zn-based oxide TFTs. As it is known, ALD offers various advantages including accurate thickness and composition control, excellent uniformity and step coverage, low defect density, low deposition temperatures, and good reproducibility [15–17]. Furthermore, due to the self-limiting and layer-by-layer (or “digital”) growth, ALD exhibits the unique in situ atomic layer doping capability of achieving precise doping with atomic level control during the deposition process.

Up to now, to our limited knowledge, the deposition of channel layers using ALD has been focused on almost pure ZnO layers. Given its low standard electrode potential, Hf is a more efficient suppressor of the generation of oxygen vacancies. Adding Hf element could suppress the growth of columnar structure, and therefore drastically decrease the carrier concentration and hall mobility in HZO films [18–20]. Hence, in this paper, Hf doped ZnO has been deposited via ALD and applied in TFTs as channel layer. The related parameters such as the Hf contents, growth temperature, channel thickness, and width/length ratio (*W* = *L*) were evaluated and optimized. The influence of Hf doping on performance of ZnO-TFT was studied. Especially, the detailed effect of Hf doping on the DOS has never been addressed. In the present letter, the DOS was calculated based on the experimentally obtained *E*_a_, which can explain the experimental observation.

## Methods

**Fig. 1:**
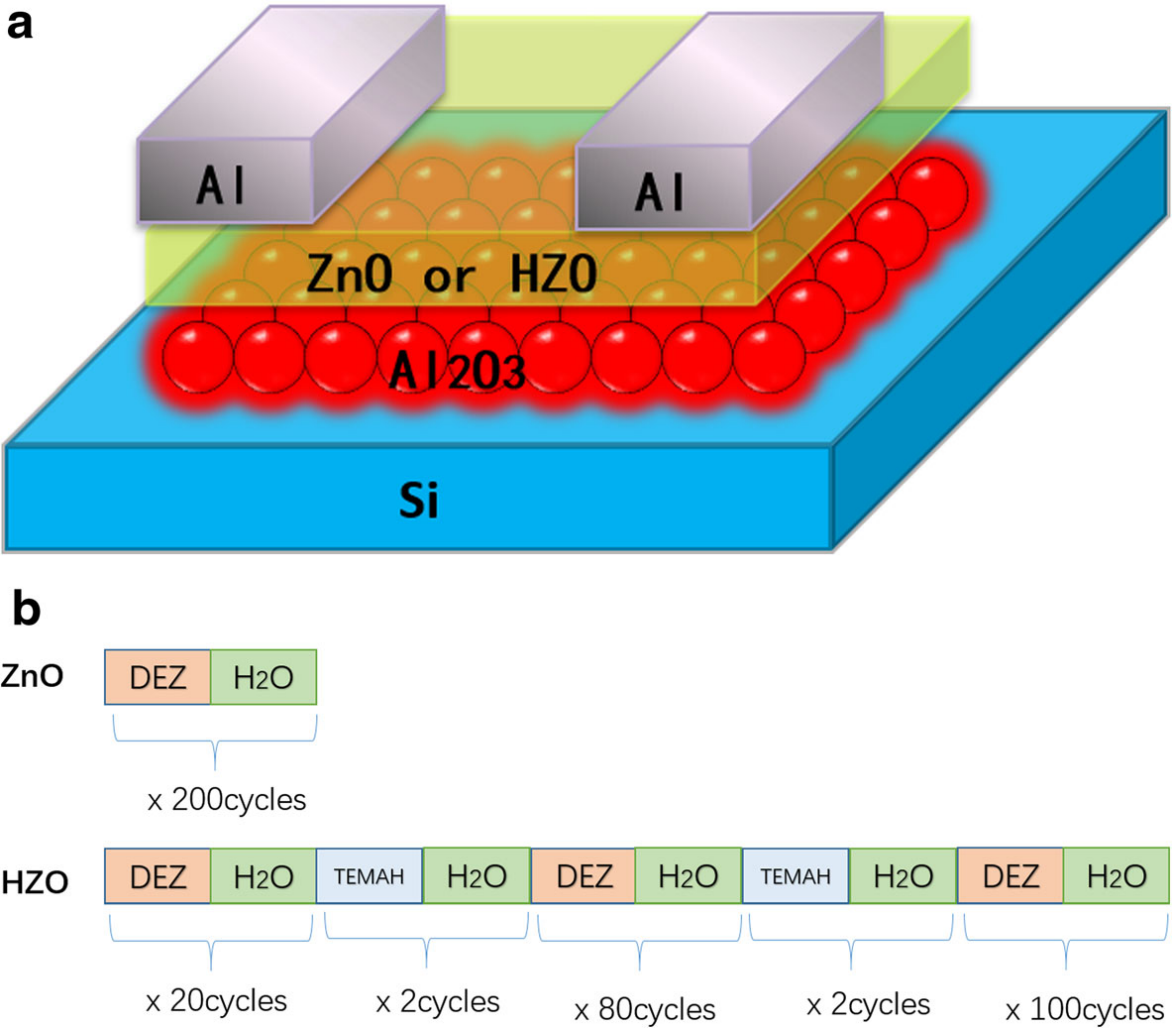
**a** Schematic structure of the devices. **b** Cycle designs of the ALD process to deposited ZnO and HZO films

Bottom-gate TFTs were fabricated on a doped n-type Si wafer as shown in Fig. 1a. Before being placed in the ALD chamber, the Si substrates were cleaned with consecutive rinses of acetone, isopropyl alcohol, and de-ionized water in an ultrasonicator for 15 min, and then treated by Ozone for 10 min. Immediately following this procedure, Al_2_O_3_ films of approximately 100-nm-thick were deposited by ALD (TFS-200) technique at 240 °C using Al(CH_3_)_3_ and H_2_O. The carried gas was nitrogen of 300 sccm. The purge/pluse time for TMA or H_2_O was 7/0.1 s. High-purity diethylzinc (DEZn), tetrakisethylmethylamino-hafnium (TEMAHf) were used as Zn, Hf source and water (H_2_O) as oxygen source at deposition temperatures of 150 °C. The detailed ALD method to deposit ZnO and Hf dop ZnO films was shown in Fig. 1b. The growth rate of ZnO and HfO_2_ films were 0.2 and 0.08 nm/cycle, respectively. Al films deposited by thermal evaporation were used for source/drain electrodes through a shadow-mask with the channel width *W* = 1000 μm and channel length *L* = 60 μm. Thermal annealing was performed in ambient air at 250 °C for 20 min. The structural property of the films was measured with X-ray diffraction measurements with Cu-Kα radiation (D/MAX). Optical transmission was measured using a double beam spectrophotometer (U-3900). The electrical properties were performed using semiconductor parameter analyzer (Agilent, 4155C) with a probe station (LakeShore, TTP4).

## Results and Discussion

**Fig. 2:**
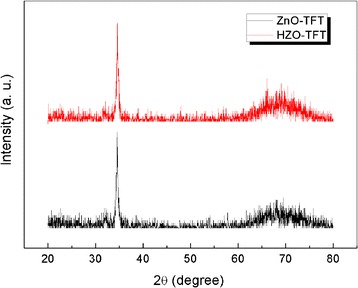
XRD patterns of ZnO-TFT and HZO-TFT on Si water

Figure 2 demonstrates the XRD patterns of ZnO-TFT and HZO-TFT on Si water. The predominant diffraction peaks correspond to the (002) orientation, which indicates that this film has a c-axis orientation polycrystalline structure regardless of Hf insertion. A similar result was obtained by Kim et al. [21]

**Fig. 3:**
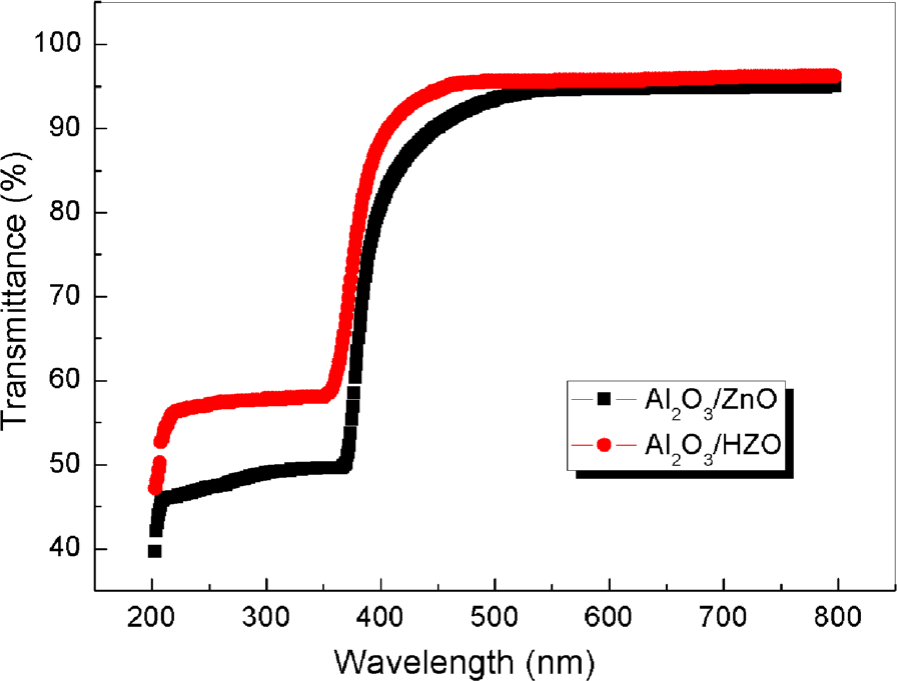
Transmittance spectra of Al_2_O_3_ (100 nm)-ZnO (40 nm) and Al_2_O_3_ (100 nm)-HZO (40 nm) deposited on glass

Figure 3 shows the transmittance spectra of Al_2_O_3_ (100 nm)-ZnO (40 nm) and Al_2_O_3_ (100 nm)-HZO (40 nm) deposited on glass at an annealing temperatures of 250 °C for 20 min. The thin films show good transparency of over 95% within the visible light wavelength range. The absorption edge shows a blue shift with the addition of Hf, consistent with the results for HfZnO prepared by other deposition techniques [22, 23].

**Fig. 4:**
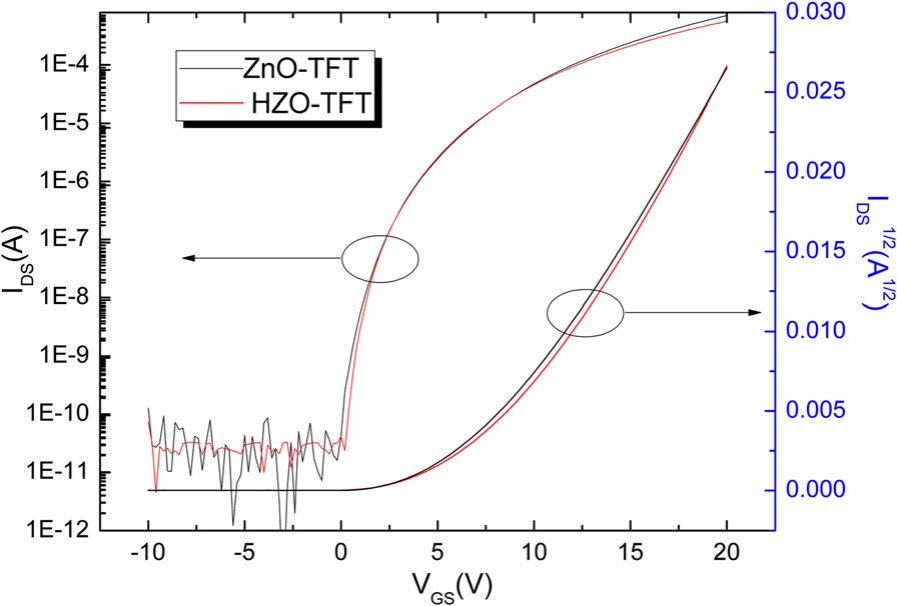
The transfer curves for the ZnO-TFT and HZO-TFT

Figure 4 shows the transfer curves of ZnO-TFT and HZO-TFT, and their related parameters are summarized in Table 1, including turn-on voltages (Von), field-effect mobility (μ), on/off ratio, and sub-threshold swing (SS). As seen in Table 1, a remarkable improvement in on/off ratio for HZO-TFT was achieved. The on/off-current ratio of the two devices were all over 10^6^, indicating that these devices have good characteristics as the backplane of OLEDs requiring a relatively high on-current and small off-current for a rapid response and low power consumption. The SS decreased from 0.4 to 0.3 V/decade. The SS is extracted at the steepest point of the log(*I*_DS_)-*V*_GS_ plot by the equation: SS = d*V*_GS_/d(log*I*_DS_). In addition, the field-effect mobility also decreased from 11.3 to 9.4 cm^2^/Vs, and *V*_on_ increased from 0 to 0.2 V by the Hf doping. These values for HZO-TFT are comparable to or even exceed the values reported by other HZO-TFTs [12, 21, 24] and much better than those fabricated by ALD [12]. In general, oxide semiconductors containing heavy metal cations with (*n* − 1) d^10^ ns^0^ (*n* ≥ 4) electronic configurations show high electron mobility as the ns^0^ orbitals are overlapped with each other. However, although Hf has a large diameter and the spherical symmetry of the ns^0^ orbitals was incorporated, the field-effect mobility and drain current decreased as the Hf content increased. This suggests that Hf ions have a higher oxygen binder energy than that of Zn ions. This can be attributed to the fact that Hf ions have a high electronegativity of 1.3. The field-effect mobility is affected by shallow traps near the conduction band and the interaction of oxygen vacancies, and zinc interstitials is an important source of n-type conductivity in ZnO [25, 26]. Therefore, suppressing the generation of oxygen-vacancy-related defects with Hf doping can effectively decrease the mobility [25]. Decrease in the SS value of HZO-TFT indicates a reduction in interface trap density.

**Table 1:** Comparison of the electrical properties of the devices

Device	*V*_on_ (V)	*μ* (cm^2^/Vs)	On/off	SS (V/decade)
ZnO-TFT	0	11.3	>10^6^	0.4
HZO-TFT	0.26	9.4	>10^7^	0.3

**Fig. 5:**
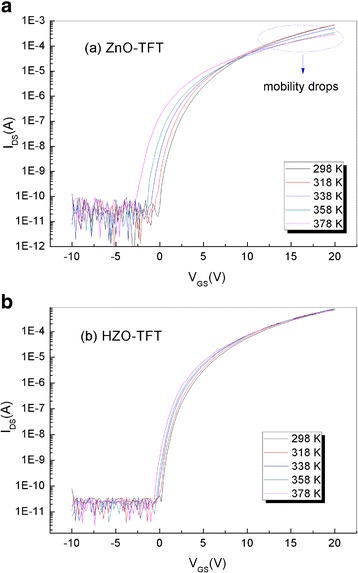
*I*_DS_-*V*_GS_ curves of **a** ZnO-TFT and **b** HZO-TFT at the different temperatures

In order to demonstrate the Hf doping on the trap density of states, the temperature-dependent field-effect measurement was used to calculate DOS. Figure 5a, b shows the *I*_DS_-*V*_GS_ curves of ZnO-TFT and HZO-TFT, respectively, as a function of the measurement temperature. The transfer measurement was carried out in the dark after the temperature had been staying at the fixed level for about 1 min. At increasing measurement temperature, all TFTs showed a negative shift of *V*_on_. It is well known for the oxide semiconductors materials that the free electrons mainly result from the detrapping from sub-bandgap trap states and the generation of oxygen vacancies [27–29]. Thermally excited electrons are detrapped from relatively shallow sub-bandgap trap states. In addition, more oxygen vacancies were induced by thermal excitation, and therefore, more free electrons were generated. The lower *V*_on_ observed at the higher temperature can be attributed to these free electrons from the sub-bandgap trap states which were generated along with the oxygen vacancies [30]. One interesting point to note from our experiment is that the mobility of ZnO-TFT decreased as the temperature increased. The drop in mobility at higher temperature is likely due to the generation of more trap states; as a result, the mobility starts decreasing. Indluru et al. have also observed a similar trend for their a-Si:H TFTs for operation temperatures higher than 348 K [31].

In the sub-threshold region, the current of the TFT devices was well described by the Arrhenius thermal activation model [32, 33]:

1 $$ {I}_{\mathrm{DS}}={I}_{D0}\cdot \exp \left[-\frac{E_a\left({V}_{\mathrm{GS}}\right)}{kT}\right] $$

where *I*_D0_ is the prefactor, *E*_a_ is the activation energy, *k* is the Boltzmann constant, and *T* is the temperature. *E*_a_ can be easily extracted by plotting log (*I*_DS_) and 1/k*T* (not shown here).

**Fig. 6:**
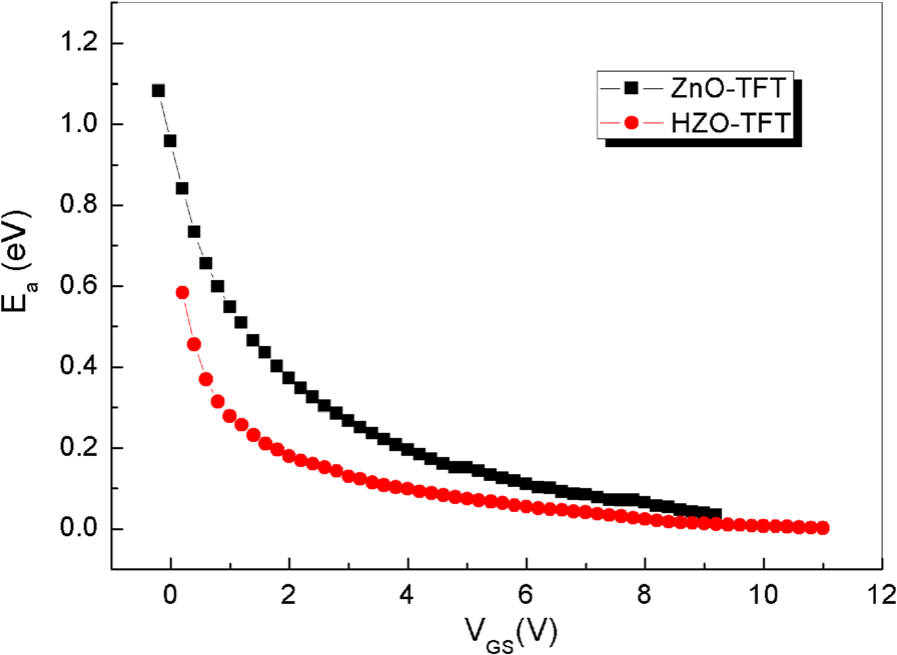
Active energy (*E*_a_) as a function of *V*_GS_

The relationship between *E*_a_ and *V*_GS_ is shown in Fig. 6. *E*_a_ (*E*_a_ = *E*_*C*_−*E*_F_) represents the energy difference between the Fermi level (*E*_F_) and the conduction band edge. The falling rates (d(*E*_a_)/d(*V*_GS_)) of ZnO-TFT and HZO-TFT are 0.59 and 0.65 eV/V, respectively. Since the falling rate of *E*_a_ with respect to V_GS_ should have the same magnitude as the rate of rising in *E*_F_, the rate-limiting process is the thermal excitation of the trapped charge. All the trap sites below *E*_F_ must be filled with electrons before any move to the *E*_F_ level in the forbidden bandgap region, so the rate of change in *E*_F_ with respect to *V*_GS_ (d(*E*_a_)/d(*V*_GS_)) is roughly inversely proportional to the magnitude of the total trap density, including the DOS of a semiconductor film (*N*_SS_) and an interfacial trap density (*N*_it_). Therefore, a much faster falling rate of HZO-TFT compared to ZnO-TFT suggests that a *N*_total_ value of HZO-TFT is reduced compared to ZnO-TFT. HZO-TFT exhibits a faster moving *E*_F_ level with respective *V*_GS_, which means the reduction in bulk and interface trap density.

**Fig. 7:**
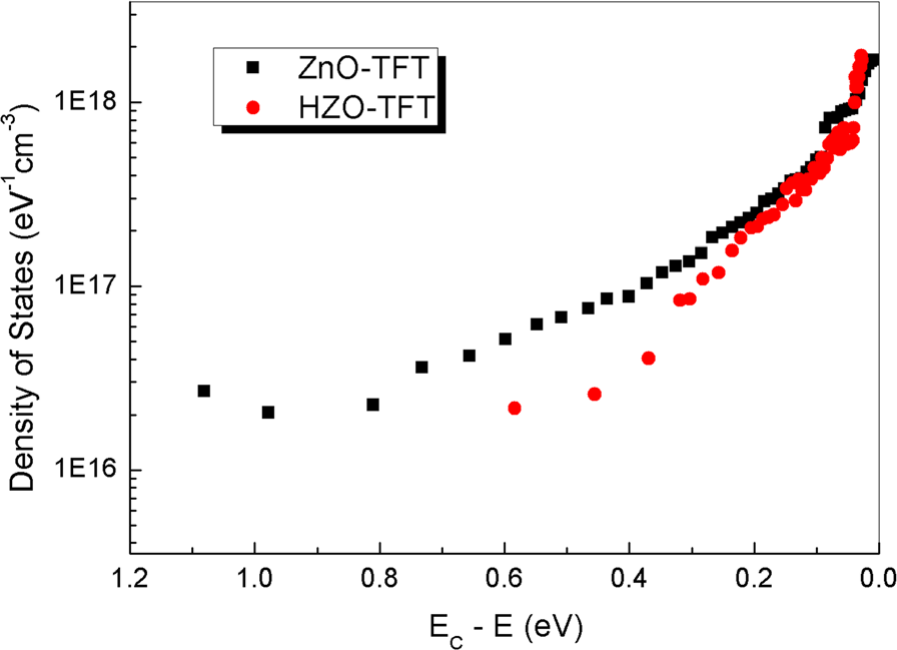
Calculated DOS distribution as a function of the energy (*E*_C_−*E*) for devices

To investigate in detail the distribution and density of tail states and deep states within energy bandgap in the proposed devices, the DOS was calculated based on the following equation [34]:

2 $$ g\left({E}_a\right)=-\frac{\varepsilon_i}{q{d}_it\frac{d{E}_a}{d{V}_{\mathrm{GS}}}} $$

where ε_*i*_ and *d*_*i*_ are, respectively, the permittivity and the thickness of the gate dielectric, *q* is the electron charge, and *t* is the thickness of the active layer. Figure 7 shows the DOS as a function of the energy (*E*_C_−*E*) for ZnO-TFT and HZO-TFT, respectively. The total DOS for HZO-TFT is much smaller than that of ZnO-TFT, which matches with its falling rates. The total DOS value at a specific energy level is the summation of *N*_it_ and *N*_SS_ because both trap states hinder the moving up of the *E*_F_ level at the interface with increasing *V*_GS_ (>*V*_FB_) [35]. Based on DOS results, we conclude that the ZnO-TFT has much more bulk traps and interface trap states than HZO-TFT. Therefore, the improved stability in HZO-TFT can be caused by the reduction in total traps causes. Using Hf doping is an effective way to suppress the generation of oxygen-vacancy-related defects and thus improve the stability of ZnO-based TFT.

**Fig. 8:**
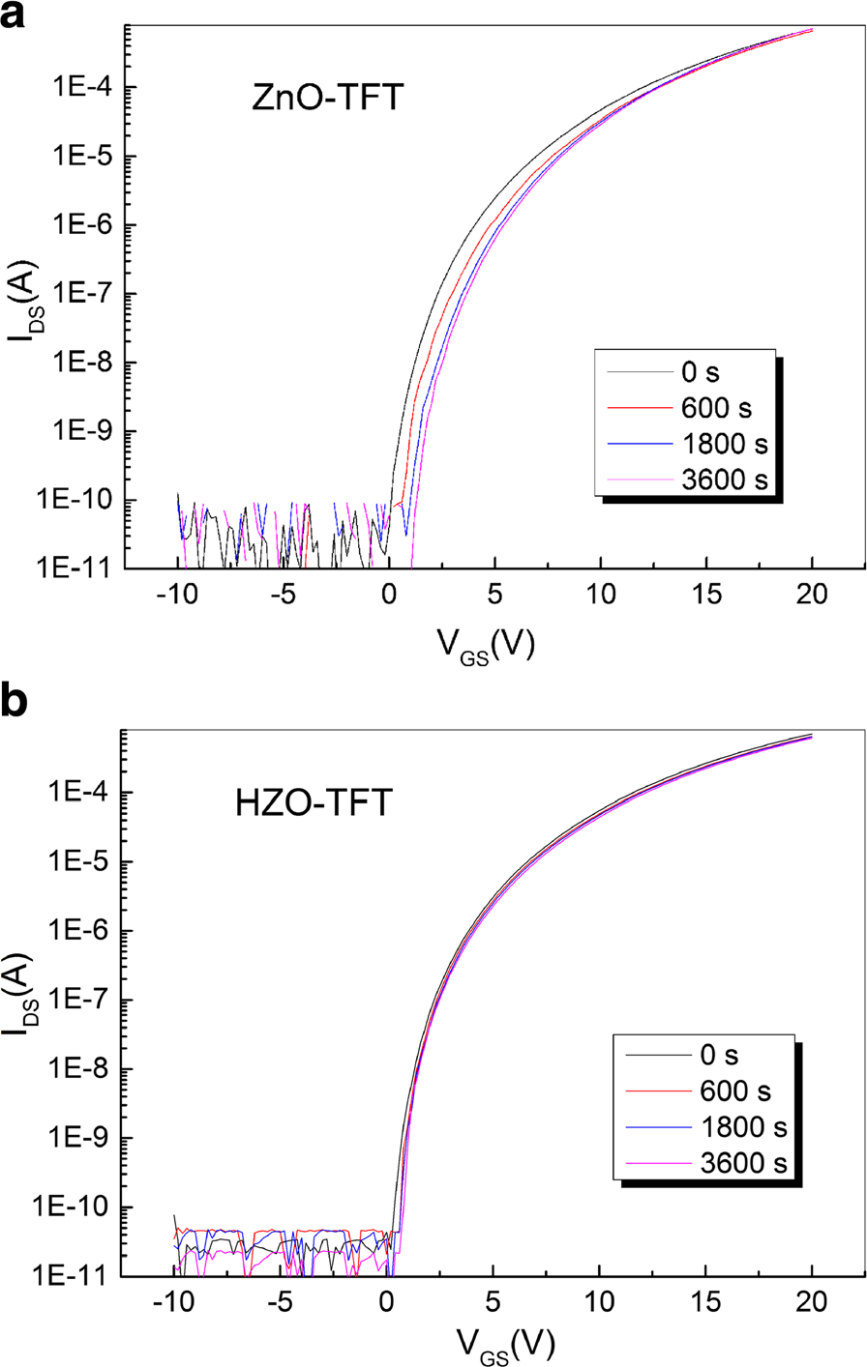
The transfer curves of **a** ZnO-TFT and **b** HZO-TFT with gate bias of 10 V stress

**Fig. 9:**
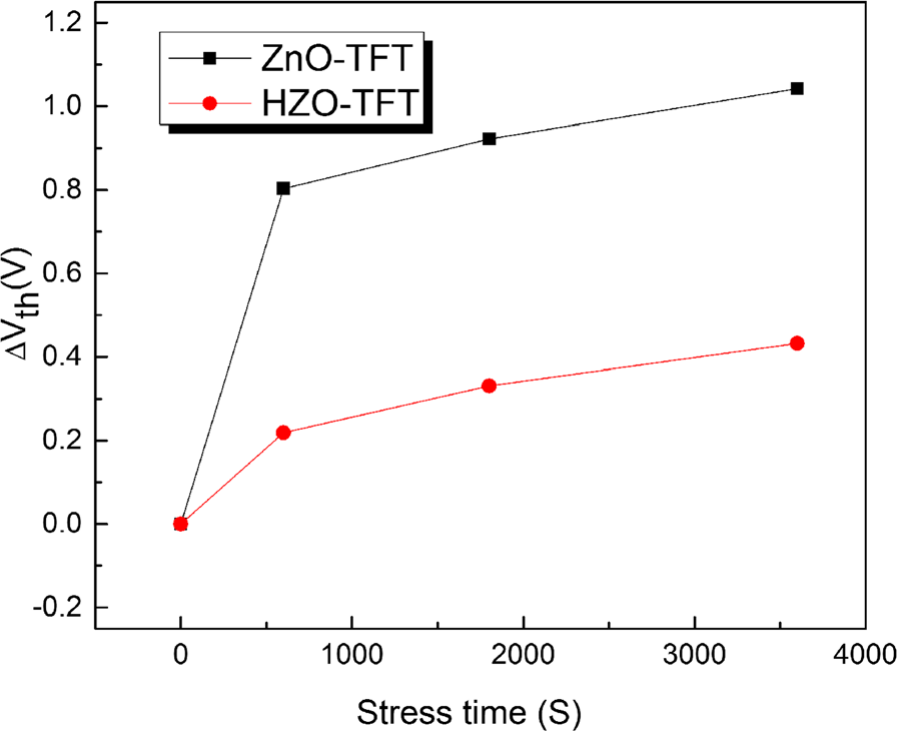
The dependence of threshold voltage shift on stress time

Figure 8 displays the transfer curves of ZnO-TFT and HZO-TFT with gate bias of 10 V stress for an hour at room temperature. Threshold voltage shifts (Δ*V*_th_) were 0.96 and 0.34 V for ZnO-TFT and HZO-TFT. HZO-TFT showed the smaller threshold voltage shift after 3600-s stress duration which indicates that it is more stable under bias stress than ZnO-TFT. Figure 9 shows the dependence of Δ*V*_th_ on the stress time. Lee reported [36] that the time dependence of Δ*V*_th_ is in agreement with a stretched-exponential equation, which can be expressed as:

3 $$ \varDelta {V}_{\mathrm{th}}\left(\mathrm{t}\right) = {V}_0\left\{1\hbox{-} \exp \left[-{\left(t/\tau \right)}^{\beta}\right]\right\} $$

where *V*_0_ = *V*_GS_−*V*_th,0_, *V*_th,0_ is the threshold voltage at the start of the stress measurement, *β* is the stretched exponential exponent, and *τ* reflects the characteristics of carrier trapping time. The obtained *τ*, *β* values are 1.58 × 10^6^ s, 0.39 for ZnO-TFT, and 2.32 × 10^6^ s, 0.56 for HZO-TFT, respectively. It demonstrates that the degradation of HZO-TFT is slower than that of ZnO-TFT under a long-time operation.

## Conclusions

In summary, we have successfully fabricated HZO-TFT via ALD, and the effect of Hf-doped ZnO on the stability of device was studied. Significantly improved on/off ratio and temperature stress stability were obtained in the HZO-TFT due to the reduced DOS, to be specific, a high field-effect mobility of 9.4 cm^2^/Vs, a suitable turn-on voltage of 0.26 V, a high on/off ratio of over 10^7^, a steep sub-threshold swing of 0.3 V/decade. The proposed HZO-TFT in this paper can act as driving devices in the next-generation flat panel displays.
