# Erratum to: The Influence of Hafnium Doping on Density-of-States in Zinc Oxide Thin-Film Fransistors Deposited Via Atomic Layer Deposition

**DOI:** 10.1186/s11671-017-1927-x

**Published:** 2017-03-07

**Authors:** Xingwei Ding, Cunping Qin, Jiantao Song, Jianhua Zhang, Xueyin Jiang, Zhilin Zhang

**Affiliations:** 1 Key Laboratory of Advanced Display and System Application, Ministry of Education, Shanghai University, 149 Yanchang Road, Shanghai, Jingan District 200070 People’s Republic of China; 2 School of Mechatronics and Automation, Shanghai University, Shanghai, 200072 China; 3 Department of Materials Science, Shanghai University, Shanghai, 200072 China

## Erratum

The original publication [1] is missing the funding information in the acknowledgement. The missing part can be found here:

“This work was funded by National Key Basic Research Program of China (2015CB655005) and Science and Technology Commission of Shanghai Municipality Program (14DZ228090).”
